# Postnatal Growth in a Cohort of Sardinian Intrauterine Growth-Restricted Infants

**DOI:** 10.1155/2017/9382083

**Published:** 2017-06-20

**Authors:** Maria Grazia Clemente, Giampiero Capobianco, Paolo Mattia Galasso, Francesco Dessole, Giuseppe Virdis, Maria Grazia Sanna, Mauro Giorgio Olzai, Lino Argiolas, Salvatore Dessole, Roberto Antonucci

**Affiliations:** ^1^Pediatric Clinic, Department of Surgical, Microsurgical and Medical Sciences, University of Sassari, Sassari, Italy; ^2^Gynecologic and Obstetric Clinic, Department of Surgical, Microsurgical and Medical Sciences, University of Sassari, Sassari, Italy; ^3^Neonatology and Neonatal Intensive Care Unit, Azienda Ospedaliero-Universitaria of Sassari, Sassari, Italy; ^4^Italian Federation of Pediatric Physicians, Rome, Italy

## Abstract

Recent studies have shown that infants with intrauterine growth restriction (IUGR) undergo catch-up growth during infancy. The aim of our study was to evaluate the postnatal growth in a cohort of IUGR infants born in a tertiary-level Obstetric University Hospital of Northern Sardinia. An observational retrospective study was conducted on 12 IUGR (group A) and 12 control infants (group B) by measuring the anthropometric parameters of weight (*W*), length (*L*) and head circumference (HC) from birth to the 3rd postnatal year. At birth, significant differences were found between group A and group B with regard to all the auxological parameters (*W*, mean 1846.6 versus 3170.8 g,* p* < 0.0001; HC, 30.1 versus 34.4 cm,* p* < 0.0001; *L*, mean 43.4 versus 49.4 cm,* p* < 0.0001). During the 1st year, 8 of 12 (70%) IUGR infants exhibited a significant catch-up growth in the 3 anthropometric parameters and a regular growth until the 3rd year of follow-up. The majority but not all infants born with IUGR in our series showed significant postnatal catch-up growth essentially during the first 12 months of life. An improved knowledge of the causes of IUGR will help to develop measures for its prevention and individualized treatment.

## 1. Introduction

A combination of environmental, genetic, and epigenetic factors, still partially unknown, can be responsible for a condition in which a fetus is unable to reach its genetically determined growth potential: this condition is defined as intrauterine growth restriction (IUGR) [1, 2]. The IUGR fetus begins to lose its growth potential during the first trimester of pregnancy, mainly as a result of uterine hypoperfusion often associated with thin umbilical cord [1, 2]. The causes can be maternal, fetal, or placental. Preeclampsia, pathologic conditions of the umbilical arteries, maternal smoking, and unbalanced diet are known risk factors of IUGR [3–6]. It is essential to diagnose IUGR by ultrasound scan before the 28th week of gestation and to monitor its evolution throughout pregnancy. In this regard, Doppler flow measurement of the fetal vessels (namely, umbilical artery, ductus venosus, and middle cerebral artery) has been found to be particularly helpful [7]. The circulatory status of the fetus is assessed especially in the middle cerebral artery, to determine the appropriate timing of delivery, that needs to be neither too early nor too late for a better outcome and prognosis [7, 8].

Clinical studies have shown that IUGR is a condition associated not only with an increased perinatal mortality, but also with significant morbidity later in life, including short stature, metabolic syndrome, and neurocognitive impairment [9–11]. The “symmetric” form of IUGR, defined by significant reduction of all anthropometric parameters including a small head circumference for gestational age, is associated with a worst prognosis compared to the “asymmetric” form of IUGR, in which the head circumference is within the normal range, and a favorable, complication-free postnatal course is generally observed [12, 13]. Among term infants, morbidity and mortality are 5-30-fold higher in low birth weight infants (LBWI) compared to infants with birth weight within the normal range (10th–90th centile) [12, 13].

The postnatal catch-up growth begins as soon as infants move to a more favorable environment and becomes evident during the first months of life. However, not all IUGR infants exhibit a postnatal catch-up growth, likely depending on the underlying causative factor/s and genetic diversity [14].

The present study reports results from a 3-year follow-up of a cohort of Sardinian IUGR infants with special emphasis on the postnatal catch-up growth.

## 2. Study Population and Methods

### 2.1. Study Population

In the year 2013, a total 27 IUGR diagnoses were made among infants born in the Gynecologic and Obstetric Clinic of the University of Sassari, Italy. Gestational age (GA) was defined on the basis of ultrasonographic estimation (Voluson E8 ultrasound system, GE Healthcare, Fairfield CT, USA) performed at the time of the first scan, as recommended (SIEOG Italian guidelines), and at about 20, 28, and 36 weeks' gestation [15]. Distributions of all measurements were similar to previously reported reference cohorts (data not reported). At the 20-week scan, details about medical history and demographic characteristics of the pregnant women were collected retrospectively. At this time, women were also informed about fetal anatomy and biometric measurements, as well as uterine and umbilical artery Doppler flow velocimetry data [15]. Ultrasonographic measurements of fetal biparietal diameter, head circumference, abdominal circumference, and femur length were performed according to standard techniques. The Hadlock equations and reference standard were used to calculate the fetal weight (EFW) centile, and EFW values less than the 10th centile defined the IUGR [15]. At the 36 week scan, pregnant women were informed about previously undiagnosed placenta praevia, severe oligohydramnios, a previously undiagnosed fetal abnormality, or noncephalic presentation [15]. Women were selected for additional, clinically indicated scans in the third trimester of pregnancy as per routine clinical care, using local and national guidelines (e.g., SIEOG guidelines). The indications for cesarean section (CS) included a not reassuring cardiotocography (85%) and a reversed end diastolic flow of umbilical artery at ultrasound evaluation (15%).

As 13 families moved out of the Sassari province and 2 newborns unfortunately deceased, the access to postnatal data was available for 12 IUGR infants (F : M = 8 : 4), enrolled as group A. Twelve term infants with a birth weight greater than 2,500 g (F : M = 8 : 4) were enrolled as a control group (group B). The parents of all the infants enrolled in this study provided informed consent.

Group A and group B newborns' main parameters are shown in Table 1, ordered by GA (column 4). Among group A, 3 of 12 infants (25%; Table 1, A1–A4) were born preterm and with a very low birth weight (VLBW), ranging from 1115 to 1400 g. The remaining group A, namely, 5 (41,6%) late preterm (Table 1, A5–A8) and 4 (33,3%) at term infants (Table 1, A9–A12), were all but one born with low birth weight (LBW), ranging from 1510 to 2450 g, and one with VLBW (1405 g).

All group A but only 2 group B infants had CS births (Table 1, B5-B6).

### 2.2. Methods

This is an observational study conducted by retrospective collection of the measures of weight (*W*), length (*L*), and head circumference (HC), at birth and at 3-month intervals during the first year, then annually in the second and third years of follow-up (*W* and *L*). All values were plotted and recorded in the growth charts as follows: (1) weight to age, (2) length to age, and (3) head circumference to age (Center for Disease Controls, Atlanta, GA, USA).

### 2.3. Statistical Analysis

Student's *t*-test was used to compare groups, considering significant a value of* p* < 0.05.

## 3. Results

At birth, significant differences were found between group A and group B infants with regard to all anthropometric parameters considered in this study (*W*, mean 1846.6 versus 3170.8 g,* p* < 0.0001; HC, 30.1 versus 34.4 cm,* p* < 0.0001; *L*, mean 43.4 versus 49.4 cm,* p* < 0.0001).

**Figure 1:**
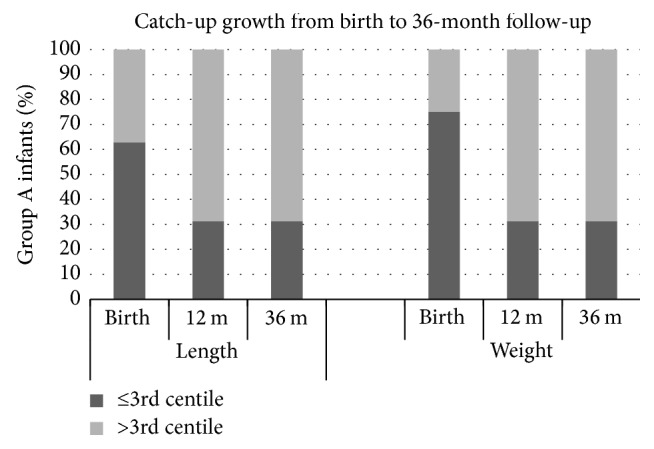
Percentage of group A infants below and above the 3rd centile cut-off for length (left panel) and weight (right panel) at birth, at 12 months (12 m) and 36 months (36 m) of age.

During the first year of life, a significant catch-up growth led to cover the differences in *L* (mean 72.6 versus 76.5 cm,* p* = ns) and to reduce those in *W* (mean 7861.0 versus 9165.0 g,* p* = 0.02) and HC (mean 43.5 versus 45.7 cm,* p* = 0.04) between the two study groups. At the age of 1 year, 8 (70%) group A infants were comparable to group B infants with respect to the 3 anthropometric parameters (Figure 1). However, analysis of data from individual patients revealed that 4 of 12 (30%) IUGR infants (Table 1, A2, A6, A8, and A10) did not exhibit catch-up growth during the first postnatal year with minimal improvement during the second and third years of follow-up (Figure 1). Categories of centiles for weight, length, and head circumference of IUGR infants at 12 months of life are reported in Tables 2, 3, and 4.

It deserves a note that among those who did not show postnatal catch-up growth, the only IUGR infant born at term (A10, Table 1) was discovered to be affected by the rare Pallister-Killian syndrome, caused by tetrasomy of chromosome 12p which is characterized by facial dysmorphism, rhizomelic limb shortness, and small hands and feet, along with corpus callosum hypoplasia. Moreover, during the postnatal years of follow-up, one of the IUGR preterm infants (A2; GA = 32 + 4; Table 1) showed failure to thrive, and it is currently under pediatric endocrinology evaluation.

## 4. Discussion

The majority of IUGR infants in our series showed significant postnatal catch-up growth during the first 12 months of life, and regular growth until 3 years of age.

Several studies in literature have reported on the postnatal catch-up growth in preterm IUGR and SGA infants, but only a few studies exist on term IUGR infants [1, 10–12].

One study conducted in North America (USA) on 42 IUGR infants has calculated growth velocity, which was significantly higher in IUGR infants compared to the control group (3.58 kg/m^2^ versus 2.36 kg/m^2^) during the first 12 months of life [13].

Another study, conducted in North Europe on 73 IUGR newborns, found catch-up growth in up to 90% of cases during the first year of life; 7% of infants among those who did not have significant catch-up growth exhibited neurological and cognitive impairment [3].

This study was not a clinical trial and was also limited by both its retrospective, observational design and the small sample size. Even with these limitations of the study, our results further confirm those reported by others. All the term IUGR infants but the one affected by Pallister-Killian syndrome exhibit a catch-up growth. More than half of the preterms IUGR did show also a significant catch-up growth, and it was significantly greater during the first 12 months of life [9].

It was not possible for us to determine for each single case whether maternal or fetal factors played a role in the development of IUGR, as well as the role played by genetic, epigenetic, and environmental factors, or likely the complex combination of multiple factors on the catch-up growth and outcome during the postnatal life.

Interesting, the recent personalized medicine approach through the Newborn Individualized Developmental Care and Assessment Program (NIDCAP) has been the focus of a study conducted on preterm infants born with severe IUGR by a multidisciplinary research working group of Harvard University [16, 17]. The NIDCAP was shown to be effective in ameliorating the neurobehavior, electrophysiology and brain structure outcomes compared to IUGR controls [16, 17]. At least 2/3 of our IUGR infants required special assistance at the Newborn Intensive Care Unit (NICU). We can therefore speculate that also our infants compromised by severe IUGR who showed postnatal catch-up growth might have had significant benefit from an individualized developmental care approach during NICU stay.

Moreover, methods of infant feeding (breast-feeding versus formula feeding) and other nutritional factors (including iron, zinc, and vitamins) might play a critical role in the catch-up growth during the first months of life [18, 19] and would deserve further, more extensive, investigation.

## Figures and Tables

**Table 1:** Main parameters of the enrolled newborns, both group A and B.

Group A infants	Sex	Delivery	G.A.	APGAR at 5′	*W* (g)	*W* (centile)	*L* (cm)	*L* (centile)	CC (cm)	CC (centile)
A1	F	CS	32	7	1115	3rd	37,0	3rd	27,0	3–10th
A2	F	CS	32 + 4	8	1300	10th	36,0	<3rd	28,0	10th
A3	M	CS	33	8-9	1400	3rd	38,0	<3rd	28,0	3rd
A4	F	CS	34	9	1405	<3rd	44,0	50th	27,5	<3rd
A5	M	CS	35	9	1583	<3rd	45,0	25th	31,5	25th
A6	F	CS	36	9	1950	3rd	42,5	3rd	31,0	3rd
A7	F	CS	36 + 3	9	2400	25th	44,5	10th	32,5	25th
A8	M	CS	36 + 6	9	1930	<3rd	43,0	<3rd	31,0	10th
A9	M	CS	37	9	2040	<3rd	43,5	<3rd	32,0	10th
A10	F	CS	37	9	1510	<3rd	42,0	<3rd	28,0	<3rd
A11	F	CS	37 + 2	9	2450	10–25th	45,3	10th	32,0	10–25th
A12	F	CS	37 + 2	9	2300	3–10th	46,0	10–25th	32,5	10th

Group B infants	Sex	Delivery	G.A.	APGAR at 5′	*W* (g)	*W* (centile)	*L* (cm)	*L* (centile)	CC (cm)	CC (centile)

B1	F	VD	38	9	2900	50th	49,0	50th	33,5	50th
B2	F	VD	38	9	3000	50th	50,0	50–75th	33,5	50th
B3	M	VD	38 + 2	9	2900	10–25th	49,5	50th	33,0	25th
B4	M	VD	39	9	3400	75th	47,0	10th	36,0	90th
B5	F	CS	39 + 2	9	2900	10–25th	48,5	25–50th	33,0	25th
B6	M	CS	40 + 2	9	3600	50th	49,0	10–25th	35,0	50th
B7	F	VD	40 + 4	9	2900	25–50th	48,5	25th	34,0	50th
B8	F	VD	41	9	2800	10th	49,5	25–50th	34,0	50th
B9	F	VD	41	9	2800	10th	48,3	10th	35,0	75th
B10	F	VD	41	9	3350	50th	50,5	50–75th	35,0	75th
B11	M	VD	41 + 1	9	3500	25–50th	51,5	50th	35,0	50th
B12	F	VD	42	9	4000	>90th	51,0	50–75th	35,5	75th

**Table 2:** Weight centile categories of IUGR (group A) infants at 12 months of postnatal life.

	Male	Female	*Total*
<3rd centile	1	3	4
3rd–50th centile	2	3	5
>50th centile	1	2	3
*Total*	4	8	12

**Table 3:** Length centile categories of IUGR (group A) infants at 12 months of postnatal life.

	Male	Female	*Total*
<3rd centile	1	3	4
3rd–50th centile	2	4	6
>50th centile	1	1	2
*Total*	4	8	12

**Table 4:** Head Circumference centile categories of IUGR (group A) infants at 12 months of postnatal life.

	Male	Female	*Total*
<3rd centile	0	3	3
3rd–50th centile	3	3	6
>50th centile	1	2	3
*Total*	4	8	12

## Abbreviations

CS: Cesarean section
EFW: Estimated fetal weight
GA: Gestational age
HC: Head circumference
IUGR: Intrauterine growth restriction
*L*: Length
LBWI: Low birth weight infants
NIDCAP: Newborn Individualized Developmental Care and Assessment Program
NICU: Newborn Intensive Care Unit
PROM: Premature rupture of the membrane
SGA: Small for gestational age
VD: Vaginal delivery
VLBW: Very low birth weight
*W*: Weight.
